# Postpartum Depression in The Arab Region: A Systematic Literature Review

**DOI:** 10.2174/1745017902016010142

**Published:** 2020-07-30

**Authors:** Khubaib Ayoub, Amira Shaheen, Shakoor Hajat

**Affiliations:** 1 Faculty of Medicine and Health Sciences, An-Najah National University, Rafidia Street, P.O.Box 7, Nablus, Palestine; 2 Department of Social & Environmental 9 Health Research (SEHR), Faculty of Public Health and Policy, London School of Hygiene and Tropical Medicine, 15- 17 Tavistock Place, London, WC1H 9SH, UAE

**Keywords:** Postpartum depression, Arab region, Public health problem, Infants, Unwanted pregnancy, Socioeconomic status

## Abstract

**Background:**

Postpartum Depression (PPD) is a major public health problem affecting mothers and their babies. However, few studies have investigated the prevalence and risk factors for postpartum depression among Arab mothers. This systematic literature review aims to determine the prevalence of PPD among mothers in Arab countries and identify the main risk factors.

**Methods:**

A review of all peer-reviewed journal published studies on PPD and its risk factors among Arab mothers until February 2016. The following data bases were searched; PubMed, Springlink, Science direct, EBSCOhost, and Arabpsychnet.

**Results:**

25 studies were included in the review. PPD rates were high in general but prevalences were close to the rates observed in other low and lower-middle-income countries. Twelve studies reported PPD prevalences in the region of 15-25%, 7 studies reported prevalences< 15% and 6 studies reported prevalences<25%. The most important risk factors for PPD were: low income and socioeconomic status, obstetric complications during pregnancy, unwanted pregnancy, ill infant, formula feeding, low social and husband support, marital and in-laws conflicts, stressful life events during pregnancy and personal or family history of depression.

**Conclusion:**

Prevalence of PPD is high in most Arab countries, with differences due in part to variations in methods of assessment. This review highlights the problem of PPD and advocates for the adoption of necessary changes in the Arab health systems such as routine screening and efficient referral systems in order to detect and treat this potentially debilitating condition.

## 1 INTRODUCTION

Postpartum Depression (PPD) is defined as the occurrence of a major depressive episode within 4 weeks of delivery [1]. Although PPD commonly begins within the first 4 weeks, onset can occur at any time during the first postnatal year, with the incidence decreasing substantially by 3 months following birth [2]

A Major Depressive Episode (MDE) is defined in the fifth edition of the Diagnostic Statistical Manual of Mental Disorders (DSM-V) [1] as the presence of at least five of the following symptoms during most of the day, nearly every day for a 2-week period with impairment, or decline of previous levels of functioning: depressed mood, often accompanied by anxiety; markedly diminished pleasure in activities; loss of appetite and weight; sleep disturbances, often insomnia; physical agitation or psychomotor slowing; fatigue and low energy; feelingsof worthlessness or inappropriate guilt; decreased concentration and decision making ability; and recurrent suicidal ideation or thoughts of death. The presence of a depressed mood or loss of interest is necessary for a diagnosis of MDE [1, 3].

PPD is a major public health problem since it affects 13% of mothers worldwide during a very sensitive period of their life, exerting a negative effect on mothers, their babies and families, and potentially their entire society [4, 5]. Different types of risk factors and predictors are found to correlate with PPD. Based on studies from Western societies, strong predictors of PPD are: depression or anxiety during pregnancy, a previous history of depression, stressful recent life events and poor social support. Less powerful or moderate predictors include: stress over childcare issues, low self-esteem and bad infant temperament such as excessive crying. Obstetric and pregnancy complications, poor partner relationship or single mothers, and low socioeconomic status and income are minor predictors of PPD, whilst no significant relationships were previously observed with race, mother’s age, education, number of births (parity) or the baby’s sex [2, 5].

Some recent studies suggest that PPD is a heterogeneous disorder with more than one distinct phenotype. These subtypes differ between the time of onset, severity of mood worsening and hopelessness, presence of anxiety, suicidal ideation and presence of pregnancy or obstetric complications [6, 7].

The prevalence of PPD is considered to be higher in low- and lower-middle income countries, where the average prevalence of depression was 15.6% (95% CI: 15.4–15.9) during pregnancy and 19.8% (95% CI: 19.5–20.0) during the postnatal period [4]. Fisher *et al*. conclude that risk factors for PPD in these countries are: low socioeconomic status; unplanned or unwanted pregnancy; younger age of the mother; being unmarried; poor family and partner relationships, lack of partner support, empathy, and practical help, having a conflict with the mother-in-law, and experiences of partner violence. History of mental health problems and giving birth to a female are also significant risk factors. However, higher education, permanent employment status and a kind trusted partner were protective factors against PPD, as well as being from the majority ethnic group in some settings [4].

Although some Arab populations were included in previous international and regional reviews [4, 8], there is no comprehensive review of postpartum depression prevalence and associated risk factors, specifically in Arab countries. The Arab nations have many features in common, including the Arabic language and cultural and traditional values as conservative societies. On the other hand, Arab countries have a great diversity in terms of socioeconomic conditions ranging from very poor countries like Somalia and Yemen to Gulf countries, which have the highest per capita income in the world [9]. This diversity and inequity continue to exist within the same community in many settings. Some of these factors may increase vulnerability to depression, and so we conducted a systematic literature review of PPD in Arab settings. This review aims to assess the prevalence of PPD in Arab countries and to ascertain risk factors for PPD in these settings.

## 2 METHODOLOGY

A comprehensive review of the peer-reviewed published literature was conducted through PubMed in February 2016 using the following terms: “postnatal depression”, “postnatal”, “postpartum depression”, “postpartum” AND”Arab”. In addition, “postnatal depression” and “postpartum depression” were combined with 22 individual Arab countries (Algeria, Bahrain, Comoros, Djibouti, Egypt, Iraq, Jordan, Kuwait, Lebanon, Libya, Mauritania, Morocco, Oman, Palestine, Qatar, Saudi Arabia, Somalia, Sudan, Syria, Tunisia, United Arab Emirates and Yemen).

This review was added to an earlier search of the same terms conducted in February 2016 in PubMed, SpringerLink, science direct, EBSCOhost and Arabpsychnet. The search engine of the Institute for Development, Research, Advocacy and Applied Care (IDRAAC), which is a search engine containing all mental health research in the Arab world from 1966 to July 2007, was also reviewed. (http://www.idrac.org.lb/) Finally, the reference lists of identified articles and the research gate website were searched manually for any further relevant articles from Arab countries.

Inclusion criteria were: 1) original studies about postpartum depression published in peer-reviewed journals; 2) concerning prevalence and/or risk factors for PPD; 3) conducted in one of the mentioned Arab countries or among Palestinians in Israel; 4) published in the English language; 5) scoring at least 5 on the methodological assessment criteria mentioned below. Exclusion criteria were: 1) MSc and PhD thesis and books; 2) Studies about treatment methods or interventions; 3) Studies about biological and genetic risk factors; 4) studies about antepartum depression unless they were about both ante- and postpartum depression; 5) Studies about immigrants or Non-Arab populations (*e.g.*, American army in Iraq); 6) Studies where the population is mixed Arab and Non-Arab; 7) Studies where we could not access the full article; and 8) review studies. One Tunisian study was originally in French but was added following translation to English in order to include more relevant studies. Fig. (**1**) shows the flow of the search process.

Two of the review authors (K.A. and A.S.) assessed the methodological quality of the retrieved papers using criteria previously developed by Mirza and Jenkins.(4, 8, 10) These criteria evaluate the studies in relation to the following points: 1) clear study objectives, 2) adequate sample size with justification (either calculated or the population size mentioned), 3) sample representative of the population, 4) clear inclusion and exclusion criteria, 5) depression measure used is reliable and valid, 6) reported response rate and/or losses explained, 7) adequate description of data, and 8) appropriate statistical analyses. Fisher *et al*. added a ninth criterion of signing a consent form.(4) Grades out of 9 were assigned to individual studies based on their fulfillment of the above criteria (Table **1**).

As the Palestinian Arab community in Israel is a large community of 1.8 million and shares the Arab and Palestinian context, another PubMed search using postpartum depression in Israel was done and yielded 68 studies. Of these, 60 were irrelevant and only 3 articles remained after the exclusion, but they were already identified from the previous search. Further manual searches retrieved additional 4 studies, but one of them was excluded as the methodology and other details were obscure.

## 3 RESULTS

A total of 88 studies about postpartum depression in Arab countries were retrieved through PubMed, 45 of them were duplicates and 22 studies were excluded after reading the abstract of the full article. Of these, 3 were exclusively about antenatal depression, 4 about interventions for PPD or studying specific hormonal or genetic predisposing factor, 5 were about non-Arab populations such as Kurdish people or the American Army in Iraq, 5 were investigating mixed Arab and non-Arab populations in Israel or comparing mixed immigrant populations, and a further 3 were a literature review, a tool validation study and a study investigating PPD among the Jordanian army. Another 2 were excluded because the full text could not be retrieved despite repeated attempts at communication with the authors.

The total number of included studies was 25 (Table **2**). Of these, 21 investigated the prevalence of depression only during the postnatal period, and 4 investigated depression during both pregnancy and after birth (Table **2**). Moreover, 24 out of 25 studies investigated probable risk factors associated with the occurrence of postpartum depression. The earliest study was published in 1997, but only 5 studies were published before 2010. Four studies were published in 2011 and 4 in 2012, but the dramatic increase was in 2014 when7 studies were published, reflecting the increased attention paid to this important condition by Arab researchers. Lastly, 3 studies were published in 2015. Six studies were conducted in Egypt, 3 studies were in each of the UAE, Saudi Arabia and Palestinians in Israel, 2 studies were from Lebanon and Morocco, and one study in each of Tunisia, Jordan, Bahrain, Qatar, Oman and Sudan.

## 4 COMPARISON OF THE USED METHODOLOGIES

The quality of the included studies, according to the previously mentioned criteria, ranged from 6 to 9 out of a maximum score of 9, reflecting the high methodological quality of these studies in general. The details of this assessment are presented in Table **2**.

### 4.1 Time and Place of Recruitment

Twenty studies recruited participants at various times during the postpartum period, and 5 recruited mothers during pregnancy and followed them at different times after birth (Table **2**), making a comparison of prevalence rates more difficult. The place of recruitment also differed between the studies. Whilst 11 studies chose to recruit mothers from hospitals(which are mostly tertiary teaching hospitals) either after giving birth or at follow up in the hospital clinics, 11 studies recruited their participants from community-based maternal and child health clinics (MCHCs) of primary health care centers (PHCCs). Lastly, 2 studies chose a combined hospital and MCHCs pool of participants and 1 study recruited mothers directly from the community by home visits. (See Table **2**).

### 4.2 Study Design

All of the 25 studies were cross-sectional prevalence studies. Seven studies used a longitudinal panel approach by measuring depression in the same participants at multiple time-points during pregnancy and the postpartum period, thus tracking the changing rates of depression in relation to the different time points. A further 3 studies used a cross-sectional approach to estimate point prevalences and a case-control design to determine risk factors. The remaining 15 studies used point or period prevalence estimates on one occasion or during a specific period after delivery (Table **2**).

### 4.3 Sample Size and Sampling Method

Only 4 studies reported a random sampling approach [10-14] and the remaining studies used convenience sampling and so their representativeness of the general population is doubtful. However, a number of studies aimed to be more representative by recruiting their sample from the entire available primary or secondary care units serving the target population [11, 15-18] rather than simply sampling the institution in which they worked. The small sample size in many studies hindered the generalization of the results.

### 4.4 Tools Used

The 25 studies used the Edinburgh Postnatal Depression Scale (EPDS) for the screening and investigation of PPD; however, different cut-off scores were sometimes used, making comparisons between studies more complicated in addition to the other methodological differences. The most used cut-off of EPDS score to screen positive for depression was ≤ 13 used by 7 studies, and a further 7 studies used a score of 10-12 to define moderate depression and ≤ 13 for severe depression. 6 studies used a cut-off of ≤ 12, 3 used ≤ 10, and one used ≤ 9. Hamdan and Tamim used both ≤ 10 and ≤ 12.

Four studies used The Mini-International Neuropsychiatric Interview (MINI) to establish PPD diagnosis after screening with EPDS [14, 16, 19, 20]. Other screening tools included the Beck depression inventory (BDI) [21], the Depression, Anxiety and Stress Scale (DASS) [17, 22], the Postpartum Depression Predictors Inventory (PDPI) [23], the Structured Interview for DSM-IV Axis Disorders- Clinician Version (SCID-CV) [24], and the Present State Examination (PSE) diagnostic protocol [25]. Regardless of the tool used, PPD rates were high in general and many studies had a prevalence close to the average rate for other low and lower-middle-income countries [4].

## 5 PREVALENCE

Prevalence of postpartum depression varied widely due to the different tools and different cut-off points used, and also because of the difference in time-points at which depression was investigated (Table **2**). Twelve studies concluded that 15-25% of mothers had or likely had postpartum depression. Seven studies reported prevalence rates less than 15% (with a minimum rate of 7%) and 6 studies reported rates> 25% (maximum 74%).

When using an EPDS cut-off score ≥ 10 (EPDS maximum score is 30), the PPD prevalence ranged from 13.2% at 6 weeks postpartum in Tunisia [26] to 43% and 44.2% in Bedouin Palestinians of Southern Israel [27] and in the Abu Dhabi hospital study [28] respectively. The rate was reported to be 73.7% in the city of kom-ombo in Egypt, but the sample size was small (57 mothers) and the time of measurement wide (2 weeks to one year postpartum) [29]. The use of this cut-off score results in high sensitivity, with a slight reduction in specificity [30]. On the other hand, the use of the more accepted cut-off score of ≥12 resulted in depression rates ranging from 9.2% in Sudan [14], 16.8% in Sharjah in UAE [16] and 17.6% in Qatar [18] to 20.1% and 21% in Morocc [19] and Lebanon [31] respectively. Bahrain was an outlier with a prevalence of 37.1% with EPDS score ≥ 12 [11]

The most recent studies used score ≤13 as a cut off for depression or used 2 cut-offs for moderate and severe depression (≤10 and ≤13). The prevalence ranged from 8-10% in Arab Palestinian mothers of Northern Israel, Saudi Arabia and Oman [15, 32, 33] to 20-22% in Jordan, Egypt, Abu Dhabi and the Palestinian mothers of Naqab [12, 13, 17, 28, 34]. The prevalence of depression assessed using diagnostic tools such as Mini-International Neuropsychiatric Interview (MINI) did not differ greatly from the results obtained by the EPDS screening tool.

## 6 RISK FACTORS

Risk factors for PPD were classified into five categories: socio-demographic factors, pregnancy and birth factors, infant-related factors, marital and family relationship factors, and psychosocial and psychological history factors. Table **3** summarizes the risk factors associated with PPD.

### 6.1 Socio-demographic Factors

The most prominent socio-demographic factor that was associated with PPD was low income or socio-economic status (SES) and it was reported to be significantly higher in depressed mothers in 7 studies. 2 studies reported an age less than 25 years as a risk factor for PPD [18, 33], whilst one reported ages older than 35 years [18] and another reported older ages at first marriage as risk factors [28]. Lower education levels were associated with PPD in 3 studies in Qatar and Naqab [18, 27, 34]. The rural residency was a risk factor in 2 studies in Lebanon and Egypt [24, 31]. The role of employment in predicting PPD was unclear. Hamdan and Tamim reported mothers’ employment as a risk factor for PPD in UAE [16], and Abdel Wahid *et al*. reported unemployment as a risk factor in Egypt [13]. Al-Hinai reported difficulties at work rather than employment itself as a predisposing factor [33]

### 6.2 Pregnancy and Birth-related Factors

The most common pregnancy and birth variable reported to be associated with PPD was an unplanned or unwanted pregnancy, as reported by 7 studies [17, 20, 24, 25, 27, 32, 34]. Primiparity was associated with PPD in 2 studies [25, 28] whilst higher parity was associated with PPD in 2 studies [16, 24]. Four studies reported complications of pregnancy or delivery such as bleeding and threatened abortion to be associated with PPD [12, 18-20] and another 4 studies reported chronic medical illnesses during pregnancy to be associated with PPD [18, 31, 35, 36]. Lastly, the association of delivery mode with PPD was inconsistent. Whilst Bener reported higher PPD in mothers undergoing Caesarean sections [18], Abdelwahid reported higher PPD among those undergoing vaginal deliveries [13]. Chaaya *et al*. reported higher PPD in urban mothers undergoing vaginal deliveries and in rural mothers undergoing Caesarean sections [31]. Bener *et al*. investigated the relationship of PPD with either history of abortion or stillbirth and history of infertility and reported both to be unassociated [18].

### 6.3 Infant Factors

Infants who were ill or of low weight predisposed their mothers to PPD in 7 studies [12, 19, 20, 24, 25, 32, 34] and two other studies found associations with premature births.(18, 36) Whilst formula feeding was higher among depressed mothers in 3 studies [16, 18, 24] breastfeeding was associated with PPD in the study by Abdelwahid *et al*. in Egypt [13]. Interestingly, having a female baby was associated with PPD in 3 Egyptian and 1 Jordanian study [13, 17, 21, 24]

### 6.4 Marital and Family Relationship Factors

A poor marital relationship and marital conflict, including physical or verbal violence and a poor or unsatisfying marital and sex life, were strongly associated with PPD in 11 studies [13, 18-20, 24-26, 32-34, 36]. Moreover, PPD was associated with poor support or practical help from the husband in 6 studies [11, 12, 20, 24, 32, 34] Conflict with the mother-in-law predisposed mothers to PPD in 4 studies [17, 18, 28, 32] whereas the absence of one’s own mothers support or conflict with her were not associated with PPD (2 studies) [11, 28].

### 6.5 Psychosocial Factors and Psychological History Factors

The most consistent predictor of PPD was a personal history of depression or mental illness, reported in 11 studies [11, 12, 23-26, 31, 32, 34-36] Stressful life events in the past year or during pregnancy were associated with higher rates of PPD in 8 studies [19, 20, 23-25, 31, 32, 34]. Moreover, poor social and family support and major financial problems or difficulty managing with income were associated with PPD in 5 [17, 18, 23, 31, 36] and 3 studies, respectively [12, 18, 20].

Interestingly, having anxiety, stress or depression during pregnancy was associated with the occurrence of PPD in 6 studies [16, 17, 23, 31, 32, 36] and the occurrence of ‘1^st^ week maternity blues’ was associated with subsequent PPD in 2 studies [23, 35]. Other factors such as perceived low parenting knowledge [17] perceived low self-efficacy [17] and dissatisfaction with overall care during pregnancy and birth were associated with PPD only in the study by Mohammad *et al*. [17]. A family history of depression was associated with higher rates of PPD in 3 studiesn [11, 32, 35].

### 6.6 Maternity Care-related Factors

 Mohammad *et al*. investigated factors related to the maternity care of the mother during labor and birth and their associations with PPD [17]. Factors found to be significantly associated with PPD at 6-8 weeks postpartum were: duration of labor of more than 11 hours; more than 8 vaginal exams; lying in lithotomy position during labor; episiotomy or requiring sutures; very painful suturing; labor more painful than expected; dissatisfaction with pain relief during labor; postpartum hemorrhage; overall poor quality of care; unhelpful doctors; mother’s desire to talk more about birth; mother not always kept informed; decisions were made without taking mother’s wishes into account; mother felt pressured to have baby quickly; mother felt labor was taken over by strangers and/or machines; doctors and midwives not encouraging nor reassuring; attendance of other mothers; mothers wanted more information during labor; mothers wanted more information about why induction was necessary; mother felt worried, anxious or frightened when labor began; mother did not feel confident in labor; mother felt out of control, frightened or helplessness.

### 6.7 Effect Size of Each Risk Factor

The earlier studies before 2012 discussed the significance of the risk factors associations with PPD based on hypothesis testing and p-values and gave no information regarding the magnitude of this association, except for the study of Chaaya*et al*. in 2002 [31]. From 2012, the authors gave more attention to the magnitude of associations by reporting odds ratios (OR). Eight of the 15 studies published in 2012 or later reported the OR of risk factors [11-13, 32-34, 36, 37], in contrast to only one [31]among 10 studies published prior to 2012. This review calculated the odds ratios of the risk factors and their 95% confidence intervals (CI), where this was possible based on the 2×2 tables presented in the reviewed papers.

Low income was the socio-demographic factor associated with the highest ORs for PPD (OR: 3.06-3.56) [12, 18, 27]. Low socio-economic status was strongly associated with PPD (OR=23.8, 95% CI [2.29-245.6]) in one study [26] and mothers who perceived their income as low were 1.5 times more likely to be depressed as reported by Al-fayoumi-Zeadna *et al*. [34]. Other socio-demographic factors such as age, residency, mother’s employment and education had odds ratios ranging from 1.3-2.5. Pregnancy and birth-related factors were reported to have odds ratios in the region 1.5-6.5. Ill or low weight infants and premature infants were 2.1-14.5 times and 1.6-3.6 times higher in their association with PPD, respectively. Similarly, formula-feeding mothers were at 1.9-5.8 higher PPD risk [16, 18]. Lastly, mothers with female babies had 2-4 times higher PPD risk compared to those having male babies in 4 studies [13, 17, 21, 24]

Poor marital relationships, including marital conflict, physical or verbal violence and unsatisfying marital and sex life, were associated with 1.5-11.7 times higher odds of depression. Poor husband support was associated with2.4-7.1 higher odds of depression in 5 studies [11, 12, 24, 32, 34]. Conflict with mother-in-law was associated with PPD in 4 studies, but only 2 reported the size of this association (OR=1.8; 2.7), respectively [32, 38]. Psychosocial variables were the most important group of PPD risk factors. Personal history of depression or mental illness led to 2 to 23 times higher odds of depression in 11 studies [11, 12, 23-26, 31, 32, 34-36], but the Qatari study which had the largest sample size (about 1400) found no association (OR=0.9) [18] Family history of depression or mental illness was 2.8 to 7.8 times higher odds of depression whilst a history of depression, anxiety or stress during the index pregnancy were associated with ORs of 2.3-14. Depressed mothers reported experiencing stressful life events in the previous pregnancy (*e.g.*, loss of a dear person or financial difficulties), with the most reported event being financial problems and difficulty managing with income.

## 7 DISCUSSION

To the best of our knowledge, this review is the largest and most comprehensive review of the prevalence and associated risk factors of postpartum depression, specifically among Arab mothers in the Arab regions. It included 25 studies from 1997 to 2015. Only studies that were conducted in Arab countries were included as Arab people in the Diaspora are likely to have different risk profiles since they experience a different cultural context.

Some studies among Arab mothers were included in previous multi-national and regional reviews. For example, Halbreich and Karkun [39] mentioned 3 Arab studies out of 143 studies worldwide, Sawyer *et al*. [8] mentioned 2 studies in their review of 35 African studies and Fisher *et al*. mentioned only one study in their review of 47 studies in low-income countries. In 2015, Haque *et al* [40]. published a review about prevalence and risk factors of PPD in the Middle East and Arab countries, and it included 15 Arab studies among 22 studies from the Middle East. Lastly, the most recent and comprehensive review was published by Alhasanat and Fry-McComish [41] in 2015 about the prevalence and risk factors for PPD among Arab women in their homelands and immigrant women in industrialized countries, regardless of their origin. The review included studies until the start of 2013 and it compared two non-homogenous populations of immigrants and non-immigrants, and concluded that the prevalence of PPD in Arab countries ranged from 10-37% and the most important risk factors were lack of social support, stressful events, partner violence and low income.

In our review, all of the included studies were cross-sectional or panel longitudinal studies. Although these types of studies are useful for reporting prevalence, causation of risk factors is less clear. In fact, 3 studies [24, 35, 37] used a case-control approach and recruited control mothers to compare risk factors for PPD. Moreover, most of these studies used convenience sampling techniques with small sample sizes, but more recent studies tended to address this by using probability sampling and reporting of sample size calculations.

Overall, published studies about postpartum depression in the Arab world are scarce and there were no identified articles in the searched databases from big Arab countries such as Algeria. The studies in other Arab countries such as Egypt and Saudi Arabia were recent (after 2010). Nevertheless, the prevalence of this problem is substantial, ranging from 8 to 40%. Most studies (12 studies) reported prevalence of 15 to 25% and 7 and 4 studies reported prevalences from 8-14% and from 26-40%, respectively. Another 2 Egyptian studies reported very high rates of 52% [23] and 74% [29], but both studies investigated small non-representative samples in a wide period of postpartum time that may include those with ‘maternity blues’ in the first 2 weeks.

The large variation in postpartum depression rates can be explained by the use of different methods and timing in the assessment of the problem (see Table **2**). Previous systematic reviews noted that the use of self-report tools results in significantly higher depression rates than interview-based assessments [5]. Moreover, higher rates in the first week after birth may be an exaggeration due to the occurrence of temporary ‘maternity blues’ in the first week postpartum [42]. Higher rates may also be due to reporting or publication bias towards higher rates and the exclusion of unpublished research reporting lower prevalence or non-significant associations. In order to overcome this obstacle, we translated a French article that met our review criteria. Nevertheless, the prevalence of postpartum depression in the Arab world seems to be higher than in the Western world [5, 43], and closer rates found in other low- and lower-middle-income countries [4]. This high prevalence was also reported in Arab countries of high income like Gulf countries. Nine of 25 studies were done in high-income countries like UAE, Saudi Arabia, Qatar, Bahrain and Oman and 5 out of 9 studies reported prevalences of 15.8% to 37.1% [11, 18, 25, 28, 37]. Three of the remaining studies were conducted in Saudi Arabia and adapted a higher cut of for EPDS ≥ 13, so prevalences were 8% (32), 10.6% (33) and 13.7% [36].

The strongest socio-demographic risk factor for PPD was low income or low socioeconomic status(OR 1.6-23.8). The effect sizes of related factors such as mother’s age, education, rural residency and mothers employment were around 2. Fisher *et al*. found that younger mother's age, lower education, low income and SES, mother's unemployment and rural or crowded areas were associated with at least a doubled PPD rate in low and lower-middle-income countries [4], whereas only low income was a significant predictor of PPD in high-income countries [5]

The presence of obstetric complications during pregnancy, such as bleeding, preeclampsia or gestational diabetes; the presence of more than one chronic disease, and unwanted pregnancies were all highly associated with higher rates of PPD, as is the case in other low-income countries [4, 8]. This could be due to the increased stress and poor mood caused by these factors. On the other hand, the mode of delivery, whether vaginal or Cesarean Section (CS) delivery, had limited associations with PPD. Although CS delivery was associated with higher PPD rates in low-income countries [4, 8], it seems that this association is based on the mother's perspective of the delivery mode [31]. This was shown in Chaaya’s *et al*. study of the differences between rural and urban postpartum depressed Lebanese women, as rural women who were delivered by CS had higher PPD rates, whilst the contrary was true among their urban counterparts [31]. The explanation given is that rural women were afraid of CS delivery and considered it as a complication of normal vaginal delivery failure [31]. This concept was emphasized in previous qualitative studies. Cesarean delivery also could be associated with longer hospital admission and subsequent separation from the family which may put further stress on rural women who usually live in proximity of their extended families compared to their big-city resident counterparts [31]

The effect of the characteristics of the new baby on mother's PPD varied. Whilst infant's ill-health, low birth weight and prematurity were associated with higher PPD rates [11, 19, 25], the birth of a female baby was only associated with PPD in Jordanian and Egyptian mothers [13, 17, 21, 24]. Although those factors predicted higher PPD rates in low-income countries(4), the birth of a female baby or a baby contrary to the desired gender were counter-intuitively not associated with PPD in Morocco [19] Bahrain [11] Qatar [18] Saudi Arabia [32, 36, 37] and Oman [33]. These results are encouraging since Arab societies are predominantly male-biased societies. Breastfeeding was investigated as a protective factor against PPD and the results were equivocal (see Table **3**), whilst formula feeding was associated with higher PPD rates [16, 18]. This may be a manifestation of the inability of depressed mothers to fulfill their mothering role and to breastfeed their infants [44]

Despite the importance of the aforementioned factors, psychosocial factors and family relationships have a deeper influence on the occurrence of PPD [8]. Poor marital relationships and marital conflict, absence of the husband, family and social support and physical and verbal abuse were predictors of higher PPD rates despite variation in assessment of these factors by either direct questions or standardized tools. These factors are recognized as PPD predictors in both high and low-income settings [4, 5, 8]. Similar to other less industrialized countries, the role of the extended family is still important as conflicts with the mother-in-law can lead to a higher incidence of PPD [4]. Although there were big differences in measuring stressful life events during pregnancy, such as the death of a dear person or major financial problems and problems of adjustment, these stresses were significantly associated with higher PPD rates. This supports the notion that PPD is mainly a psychosocial problem [3, 5, 45] In addition, the personal history of depression or mental illness or depression during the index pregnancy increased the risk of PPD occurrence. Although the Bahraini study found antepartum depression to be unassociated with PPD, it did not indicate how depression was measured during pregnancy. It seems that participants were only asked if they had a history of depression during pregnancy as the study is performed at two months postpartum. Those who found a relationship between antepartum and postpartum depression measured depression in a standardized way during pregnancy, hence their results are more informative and correspond more with international studies [4, 5].

Mohammad *et al*. found a significant association between the quality and satisfaction of the maternity care offered during delivery and subsequent development of PPD. The conclusion was that PPD is associated with negative experiences of the mother during giving birth, such as feeling out of control, lacking appropriate knowledge, being uninformed about obstetric decisions and more invasive obstetric interventions [17]. Since there was no objective assessment of maternity services, it is difficult to determine whether negative birth experiences may predispose to psychological problems, or alternatively, whether it is the depression that causes mothers to recall birth experiences badly.

In addition to the difference in investigation time and tools, the variation in PPD prevalence and reported risk factors could be explained in the context of the new studies calling to consider PPD as a heterogeneous condition with more than one characteristic subtype that differs in the time of presentation, severity and associated risk factors [6, 7]. There could be different subtypes of PPD among Arab women, but this needs more in-depth, robustly designed studies rather than simple cross-sectional assessments.

From the available evidence, it seems that low income and socio-economic status; obstetric complication during pregnancy; unwanted or unplanned pregnancy; ill newborn; formula feeding; poor marital relationships and low social support; personal or family history of depression or mental illness; antepartum depression; and stressful life events during pregnancy are the main predictors of PPD in the Arab people. The high prevalence of postpartum depression in the Arab Countries should raise concerns about screening efforts during pregnancy and postpartum periods using simple but valuable tools like EPDS. More focus should be centered on those at higher risk of developing PPD, including those with antepartum depression and poor marital and social support, lower-income and those with a history of depression. Maternal and Child Health clinic staff should work closely with mothers to identify high-risk mothers and to provide the needed support and education as an integral part of the provided health care.

The limitations of this study include the lack of complete information in some studies despite the authors’ efforts to contact the study authors, with some not replying and others no longer possessing the data. This made the calculation of many odds ratios and meta-analysis of odds ratios problematic. Moreover, the difference in timing and tools used to assess prevalence precluded a good meta-analytical approach to determine overall PPD rates.

## CONCLUSION

In conclusion, published research on PPD in the Arab world is still scarce, despite the fact that PPD is high among Arab women and is likely to affect at least 1 in 5 mothers. In order to avoid “maternity blues”, it is recommended to screen for PPD after the first week of giving birth. Furthermore, health care services should start to introduce psychological support in the treatment plan for women who present with obstetric complications during pregnancy. It is crucial for researchers and policymakers to target this area with appropriate tools and approaches in order to avoid the deleterious effects PPD can have on mothers, children, families, and societies.

## Figures and Tables

**Fig. (1):**
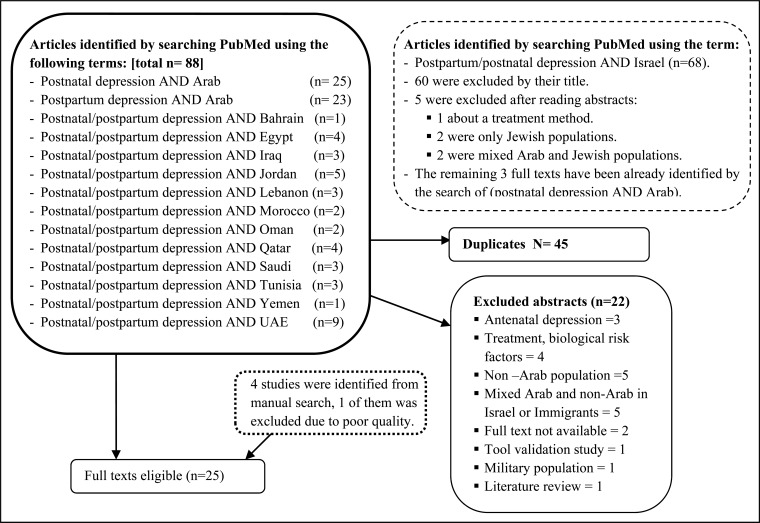
Schematic diagram of the literature search process.

**Table 1:** Methodological quality assessment of the studies about postpartum depression in Arab countries. (1 = yes; 0 = no)

**Study**	**Clear study aims**	**Adequate sample size or justification**	**Representative sample (with justification)**	**Clear inclusion & exclusion criteria**	**Measure of mental health valid**	**Response rate reported & losses given**	**Adequate description of data**	**Appropriate statistical analysis**	**Appropriate informed consent **	**Total score**
**Ghubash&Abou-Saleh (1997)**	1	0	0	1	1	1	1	1	1	**7**
**Chaaya *et al*. (2002)**	1	1	1	1	1	0	1	1	1	**8**
**Agoub *et al* (2005)**	1	0	0	1	1	0	1	1	1	**6**
**Green *et al* (2006)**	1	0	0	1	1	1	1	1	1	**7**
**Alami *et al* (2006)**	1	0	0	1	1	1	0	1	1	**6**
**Masmoudi *et al*. (2008)**	1	1	0	1	1	0	1	1	1	**7**
**Hamdan & Tamim (2011)**	1	0	1	1	1	1	1	1	1	**8**
**Glasser *et al*. (2011)**	1	0	0	1	0	1	1	1	1	**6**
**Mohammad KI *et al* (2011)**	1	1	1	1	1	1	1	1	1	**9**
**Mohamed NA *et al*. (2011)**	1	0	1	1	1	0	0	1	1	**6**
**Glasser *et al* (2012)**	1	1	1	1	1	0	1	1	1	**8**
**Al Dallal & Grant (2012)**	1	1	1	1	1	1	1	1	1	**9**
**Bener *et al*. (2012)**	1	1	1	1	1	1	1	1	1	**9**
**Abdelwahid *et al*. (2012)**	1	1	1	1	1	1	1	1	1	**9**
**Saleh *et al*. (2013)**	1	1	1	1	1	1	1	1	1	**9**
**Alharbi & Abdulghani (2014)**	1	1	1	1	1	0	1	1	1	**8**
**Alasoom & Koura (2014)**	1	1	0	0	1	0	1	1	1	**6**
**Al Hinai (2014)**	1	1	1	1	1	0	1	1	1	**8**
**Hassanein *et al*. (2014)**	1	1	1	1	1	1	0	1	1	**8**
** El-Hachem *et al* (2014)**	1	1	1	0	1	1	1	1	1	**8**
**Mohammed ES *et al*. (2014)**	1	1	1	1	1	1	1	1	1	**9**
**Mohamed H *et al*. (2014)**	1	0	0	1	1	0	1	1	1	**6**
**Alfayumi-Zeadna *et al*. (2015)**	1	1	0	0	1	1	1	1	1	**7**
**Al-Modayfer *et al*. (2015)**	1	1	1	0	1	1	1	1	1	**8**
**Khalifa *et al*. (2015)**	1	1	1	1	1	1	0	1	1	**8**

**Table 2:** Summary of the included studies with prevalence and tool used.

**Study & year**	**Country**	**Quality**	**No. of participants**	**Non-Response** **rate**	**Follow up drop rate**	**Recruitment Setting**	**Study design**	**Time of investigation**	**Tools used**	**Cut-off**	Prevalence [95% CI]*
**Ghubash&Abou-Saleh (1997)**	**UAE, Dubai**	**7**	**95**	**23.8%**	**-**	**Tertiary hospital**	**Longitudinal**	**2^nd^ day postpartum** **7^th^ day postpartum** **8 ±2 wks postpartum** **30 ±2 wks postpartum**	**SRQ** **EPDS** **PSE** **=**	**-** **≥ 12** **-**	**24.5% [16.26% - 34.28%]** **17.8% [11.06% - 27.38%]** **15.8% [9.4% - 25.03%]** **4.2% [1.36 %- 11.04%]**
**Chaaya *et al*. (2002)**	**Lebanon, Beirut and Bekaa Valley**	**8**	**396** **[207 urban** **+189 rural]**	**-**	**26%** **32% urban** **20% rural**	**9 hospitals**	**Cross-sectional**	**3-5 mo**	**EPDS**	**≥ 12**	**21% [17.12% - 25.37%]** **Rural:26%[19.97%-32.89%]** **Urban: 16% [11.37%-21.8%]**
**Agoub *et al* (2005)**	**Morocco, Casablanca**	**7**	**144**	**-**	**-**	**MCH clinic**	**Longitudinal**	**2-3 wks postpartum** **6 wks postpartum** **6 mo postpartum** **9 mo postpartum**	**EPDS** **MINI** **MINI** **=** **=**	**≥ 12** **-** **-**	**20.1% [14.11% - 27.81%]** **18.7% [12.92% - 26.29%]** **6.9% [3.56% - 12.73%]** **11.8% [7.23% -18.5%]** **5.6%[2.61% -11.02%]**
**Green *et al* (2006)**	**UAE, Abu Dhabi**	**7**	**125**	**10%**	**32% at 3mo** **55% at 6mo**	**Hospital**	**Longitudinal**	**3mo** **6mo**	**EPDS** **=** **EPDS** **=**	**≥ 13** **10-12** **≥ 13** **10-12**	**22.1% [14.14% -32.58%]** **22.1% [14.14% -32.58%]** **12.5% [5.59% - 24.69% ]** **19.6% [10.66% - 32.83%]**
**Alami *et al* (2006)**	**Morocco, Casablanca**	**7**	**100**	**18.6%**	**-**	**MCH clinic**	**Longitudinal**	**2-3wk postpartum** **12 wk postpartum** **24 wk postpartum** **36 wk postpartum** **-**	**MINI** **=** **=** **=** **EPDS**	**≥ 13**	**16.8% [10.40% - 25.92%]** **14%[8.14% - 22.71%]** **12% [6.63% - 2.04%]** **6% [2.46% - 13.12%]** **21% [13.75% - 30.53%]**
**Masmoudi *et al*. (2008)**	**Tunisia, Sfax**	**6**	**213**	**-**	**36.15%**	**Tertiary hospital**	**Longitudinal**	**1^st^wk postpartum** **6-10 wks postpartum**	**EPDS** **=**	**≥ 10**	**19.2% [14.31% - 25.32%]** **13.2% [8.25% - 20.38%]**
**Hamdan & Tamim (2011)**	**UAE, Sharjah**	**9**	**137**	**16.66%**	**8.66%**	**MCH clinic**	**Longitudinal**	**2 mo postpartum**	**MINI** **EPDS** **=**	**-** **≥ 10** **≥ 12**	** 10.1% [5.91%- 16.85%]** **16.8% [11.15% - 24.34%]** **10.8% [6.47% - 17.71%]**
**Glasser *et al*. (2011)**	**Bedouin Palestinian Arabs in Israel**	**6**	**104**	**6.3%**	**-**	**MCH clinic**	**Cross-sectional**	**within 3mo of birth**	**EPDS in Hebrew**	**≥10** **≥13**	**43% [33.7% - 53.34%]** **26% [18.08% - 35.65%]**
**Mohammad KI *et al* (2011)**	**Jordan, Irbid**	**9**	**353**	**33.1%**	**-**	**Teaching hospital and** ** 5 MCH clinics**	**Longitudinal**	**6-8 wks** **6 mo**	**EPDS** **=** **DASS-21**	**≥ 13** **-**	**22.1% [17.95% - 26.87%]** **21.2% [17.17% - 25.97%]** **-**
**Mohamed NA *et al*. (2011)**	**Egypt, Asiut**	**6**	**110**			**Family planning clinic of private hospital**	**Cross-sectional**		**EPDS** **PDPI**	**≥ 13** **-**	**51.8% [42.6%- 60.9%]** **-**
**Glasser *et al* (2012)**	**Palestinian Arabs in Northern Israel**	**8**	**2,326**	**-**	**-**	**58 MCH clinic**	**Cross-sectional**	**6 wks postpartum**	**EPDS** **=**	**≥ 10** **≥ 13**	**16.3% [14.82% - 17.87%]** **8% [6.95% - 9.2% ]**
**Al Dallal & Grant (2012)**	**Bahrain**	**9**	**237**	**5.2%**	**-**	**20 PHC center + 2 clinics**	**Cross-sectional**	**8 wks postpartum**	**EPDS** **=**	**≥ 12**	**37.1% [31.03% - 43.66%]**
**Bener *et al*. (2012)**	**Qatar**	**9**	**1,397**	**27.4%**	**-**	**12 PHC centers (9 urban & 3 semi-urban)**	**Cross-sectional**	**during first 6mo postpartum**	**EPDS** **DASS-21**	**≥ 12** **-**	**17.6% [15.67% - 19.73%]** **18.6%**
**Abdelwahid *et al*. (2012)**	**Egypt, Ismailia, El Mahsama village**	**9**	**200**	**5%**	**-**	**2 family practice centers**	**Cross-sectional**	**6-8 wks postpartum**	**EPDS**	**≥ 13**	**22% [16.3% - 27.7%]**
**Study & year**	**Country**	**Quality**	**No. of participants**	**Non-Response** **rate**	**Follow up drop rate**	**Recruitment Setting**	**Study design**	**Time of investigation**	**Tools used**	**Cut-off**	Prevalence [95% CI]*
**Saleh *et al*. (2013)**	**Egypt, Mansoura**	**9**	**379**	**-**	**11.7%**	**University Teaching Hospital**	**Cross-sectional then Case control**	**1 wk** **1,3,12 mo postpartum**	**SCID-CV** **EPDS**	**-** **≥ 13**	**One year prevalence** **17.9% [14.4% - 22.2%]**
**Alharbi & Abdulghani (2014)**	**Saudi Arabi, Riyadh**	**8**	**352**	**-**	**-**	**University & Military Hospitals + Vaccination PHC Centers**	**Cross-sectional then Case control**	**8–12 wk postpartum**	**EPDS **	**≥ 10**	**33.2% [28.5%- 38.3%]**
**Alasoom & Koura (2014)**	**Saudi Arabia, Dammam**	**6**	**450**	**-**	**-**	**5 vaccination PHC centers**	**Cross-sectional**	**2-6 months postpartum**	**EPDS**	**10-12 / ≥ 13**	**Moderate: 9.8% /Severe: 8%** **Total: 17.8% [14.5% -21.5%]**
**Al Hinai (2014)**	**Oman, Al-Dakhliya governorate**	**8**	**282**	**-**	**-**	**Postnatal and vaccination PHC clinics**	**Cross-sectional**	**2 weeks postpartum** **8 weeks postpartum**	**EPDS**	**10-12/ ≥ 13** **10-12/ ≥ 13**	**15.2% / 13.5% ** **13.8%/ 10.6%**
**Hassanein *et al*. (2014)**	**Egypt, Suhag**	**8**	**290**	**0**	**-**	**Family planning clinics of university hospital**	**Cross-sectional**	**within 3 months postpartum**	**EPDS** **BDI**	**>13** **Mild, moderate, severe**	**39% [33.5%- 44.7%]** **31%** **25.7%** **43.4%**
** El-Hachem *et al* (2014)**	**Lebanon, Beirut.**	**8**	**228**	**6.9%**	**6.6%**	**University hospital **	**Cross-sectional** **then Case control**	**2^nd^ day** **day 30-40**	**EPDS** **=**	**≥ 9** **≥ 9**	**33.3% [27.5%- 39.7%]** **12.8% [8.3% - 19%]**
**Mohammed ES *et al*. (2014)**	**Egypt, Al-Minia, El-Burgaia village.**	**8**	**200**	**5.6%**	**-**	**Community study**	**Cross-sectional**	**within 14 months of delivery**	**EPDS**	**10-12 / ≥ 13**	**29.5%/ 20%** **Total: 49.5% [42.6%- 56.3%]**
**Mohamed H *et al*. (2014)**	**Egypt, Kom-ombo City, Aswan**	**6**	**57**	**-**	**-**	**General hospital, MCH and private clinics **	**Cross-sectional**	**2 wk to 1 yr postpartum**	**EPDS**	**≥ 10**	**73.7% [61%- 83%]**
**Alfayumi-Zeadna *et al*. (2015)**	**Palestinian Arabs in Israel, Naqab**	**7**	**564**	**2.7%**	**-**	**8 MCH clinics**	**Cross-sectional**	**4 weeks to 7 months** **postpartum**	**EPDS**	**≥ 10** **≥ 13**	**31% [27.2%- 34.8%]** **19.1% [16.1% - 22.6%]**
**Al-Modayfer *et al*. (2015)**	**Saudi Arabia, Riyadh**	**8**	**571**	**52.4%**	**-**	**Major Hospital**	**Cross-sectional**	**5 weeks postpartum**	**EPDS**	**≥ 13**	**13.7% [11.1%- 16.7%]**
**Khalifa *et al*. (2015)**	**Sudan, Khartoum**	**8**	**238**	**57.1%**	**20.6%**	**Clinics of 2 major public tertiary hospitals**	**Cross-sectional**	**3 months postpartum**	**EPDS** **MINI**	**≥ 12** **-**	**9.2% [6.2% - 13.6%]** **-**

*95% Confidence Interval (CI) was calculated using the formula: [p±(z√(pq/n)+1/(2n))].** SRQ:** Self-Report Questionnaire. **PSE:** Present State Examination tool. **MINI:** Mini International Neuropsychiatric Interview.** EPDS:** Edinburgh Postnatal Depression Scale. **BDI:** Beck Depression Inventory. **SCID-CV:** Structured Interview for DSM-IV Axis Disorders, Clinician Version. **PDPI:** Postpartum depression predictors inventory. **DASS-21:** Depression Anxiety and Stress Scale

**Table 3:** Factors identified to be associated with postpartum depression.

**Variable**	**Study**	**Associated factor**	**OR [95% CI]**
**Socio-demographic variables**			
Age	Bener (2012)	Age > 35y vs. 25-35y; Age<25y vs. 25-35y.	1.96 [1.43-2.96] 2.22 [1.48-3.33]
	Alhinai (2014)	Age < 25 y	-
	Green (2012)	Older age at first marriage	-
Education	Glasser (2011)	≤10 years vs. High school	1.29 [0.48-3.45]
	Bener (2012)	Illiterate Primary education Intermediate education	2.21 [1.33-3.70] 2.10 [1.39-3.19] 1.65 [1.09-2.50]
	Al-fayumi-Zeadna (2015)	Low level of education	1.6 [1.1–2.4]
Residency	Chaaya (2002)	Rural vs. Urban	1.83 [1.12-2.99]
	Saleh (2013)	Rural	1.23 [0.59- 2.57]
Mother’s employment	Hamdan (2011)	Employed	2.55 [0.77-8.35]
	Abdelwahid (2012)	Unemployed (housewife)	3.4 [1.04-10.9]
	Alhinai (2014)	Work difficulties	2.4 [1.42-4.08]
Low Income or Socioeconomic status (SES)	Masmoudi (2008)	Low SES	23.8 [2.29-245.6]
	Glasser (2011)	Low income	3.46 [1.18-10.13]
	Mohamed NA (2011)	Low SES	-
	Bener (2012)	Low income	3.06 [2.06-4.55]
	Saleh (2013)	Low SES	t = −2.06; p<0.05^*^
	Al-fayumi-Zeadna (2015)	Perceived low income	1.6 [1.1–2.5]
	Mohammed ES (2014)	Low income	3.56 [2.15- 5.89]
**Pregnancy and birth variables**			
Parity	Ghubash (1997)	Primiparity (first child)	5.25 [1.51-18.25]
	Green (2006)	Primiparity	-
	Hamdan (2011)	≥ 1 child	4.53 [0.97-21.16]
	Saleh (2013)	Higher parity	3.27 [1.18-9.07]
Obstetric complications during pregnancy	Agoub (2005)	Complication during pregnancy	6.42 [1.59-25.82]
	Alami (2006)	Complication during pregnancy	6.66 [1.53-28.97]
	Bener (2012)	Complication during pregnancy	1.79 [1.34-2.40]
	Mohammed ES (2014)	Complication after delivery	2.84 [1.18-6.81]
Medical disease during or before pregnancy	Chaaya (2002)	≥ 2 problems	2.42 [1.13-5.15]
	Bener (2012)		-
	El-Hachem (2014)		5.28 [1.66- 16.78]
	Al-Modayfer (2015)		2.96 [1.58- 5.55]
Unwanted or unplanned pregnancy	Ghubash (1997)		3.09 [0.35-102]
	Alami (2006)		0.86 [0.31-2.35]
	Glasser (2011)		2.30 [1.04-5.08]
	Mohammad KI (2011)		-
	Saleh (2013)		8.14 [2.25-25.49]
	Alasoom (2014)		2.1 [1.24-3.55]
	Al-fayumi-Zeadna (2015)		1.6 [1.0–2.5]
Delivery mode	Chaaya (2002)	CS in rural & vaginal y in urban areas	6.58 [1.25-34.76]
	Bener (2012)	Caesarean delivery (CS)	1.40 [1.04-1.87]
	Abdelwahid (2012)	Normal delivery	3.9 [1.1-6.7]
**Infant variables**			
Infant is ill or low weight	Ghubash (1997)	Ill infant	3.83 [1.06-13.76]
	Agoub (2005)	Low weight	14.5 [1.44-145.4]
	Alami (2006)	Low weight	0.99 [0.39-2.48]
	Saleh (2013)		2.40 [1.03-5.57]
	Alasoom (2014)		2.09 [1.14- 3.86]
	Al-fayumi-Zeadna (2015)		3.9 [1.2–12.8]
	Mohammed ES (2014)		-
Premature infant	Bener (2012)		1.64 [1.06-2.54]
	Al-Modayfer (2015)		3.6 [1.12–11.61]
Female infant (baby gender)	Mohammad KI (2011)		-
	Abdelwahid (2012)		3.9 [1.3-12.4]
	Saleh (2013)		2.25 [1.08-4.68]
	Hassanein (2014)	Having 2 females	1.97 [1.22- 3.2]
Infant feeding	Hamdan (2011)	Formula	5.83 [1.81-18.79]
	Bener (2012)	Formula	2.58 [1.72-3.86]
	Abdelwahid (2012)	Breastfeeding	3.8 [1.3-11.4]
	Saleh (2013)	Bottle feeding	1.92 [0.92-3.99]
**Marital and family relationship variables**			
**Poor marital relationship or marital conflict (including physical or verbal violence, Unsatisfying marital and sexual life)**	Ghubash (1997)	Conflict during pregnancy Previously divorced mother	10.35 [2.78-38.5] 5.45 [1.26-23.46]
	Agoub (2005)		3.16 [1.64-25.05]
	Alami (2006)		0.61 [0.18-2.05]
	Masmoudi (2008)		0.90 [0.23-3.42]
	Bener (2012)	Poor marital relationship	1.13 [1-1.29]
	Abdelwahid (2012)	Poor husband relationship	12 [4.2-34.5]
	Saleh (2013)	Negative attitude toward spouce	8.04 [3.42-18.92]
	Alassom (2014)		11.74 [5.15-26.32]
	Alhinai (2014)	Conflict with family member	1.7 [1.10-2.67]
	Al-fayumi-Zeadna (2015)		1.5 [1.9–2.2]
	Al-Modayfer (2015)	Poor perception of marital relationship	6.53 [1.29- 32.97]
Poor husband support or practical help	Alami (2006)		-
	Al-Dallal (2012)		2.41 [1.24-4.69]
	Saleh (2013)		7.1 [3.16- 15.9]
	Alasoom (2014)		2.5 [1.24-5.03]
	Al-fayumi-Zeadna (2015)		2.6 [1.3–4.9]
	Mohammed ES (2014)	Supportive husband is protective	0.18 [0.05- 0.74]
Conflict with mother-in-law	Green (2006);		-
	Mohammad KI (2011)		-
	Bener (2012)		1.79 [1.33-2.42]
	Alasoom (2014)		2.72 [0.81- 9.14]
**Psychosocial variables**			
History of depression or mental illness	Ghubash (1997)		10 [2.53-39.49]
	Chaaya (2002)		1.79 [0.87-3.74]
	Masmoudi (2008)		23.4 [2.28-239.6]
	Al-Dallal (2012)		8.13 [3.77-17.5]
	Mohamed NA (2011)		-
	Saleh (2013)		t = 2.19; p< 0.05^*^
	Alasoom (2014)		2.1 [1.2-3.64]
	Al-fayumi-Zeadna (2015)	History of emotional problems	3.2 [1.7–5.8]
	El-Hachem (2014)		6.80 [1.65-28.04]
	Mohammed ES (2014)		-
	Al-Modayfer (2015)		3.86 [2.09- 7.12]
Family history of depression	Al-Dallal (2012)		2.77 [1.03-7.46]
	Alasoom (2014)		3.1 [1.26-7.8]
	El-Hachem (2014)		7.78 [2.46- 24.62]
Ante-partum depression, anxiety or stress	Chaaya (2002)		6.66 [3.47-12.78]
	Hamdan (2011)	Depression during 2^nd^ trimester; Depression during 3^rd^ trimester	7.05 [1.21-40.90] 14.1 [1.88-105.5]
	Mohammad KI (2011)	Stress and depression	-
	Mohamed NA (2011)	Depression and anxiety	-
	Alasoom (2014)	Prenatal anxiety/prenatal depression	2.33 [1.12- 4.85]
	Al-Modayfer (2015)	Stress during pregnancy	2.75 [1.69- 4.50]
Maternity blues	Mohamed NA (2011)		-
	El-Hachem (2014)		1.23 [1.11- 1.36]
Stressful life events (in the past year or during pregnancy)	Ghubash (1997)		14.71 [1.84-117]
	Chaaya (2002)		1.19 [0.63-2.27]
	Agoub (2005)		8.31 [3.26-21.15]
	Alami (2006)		0.79 [0.32-1.97]
	Mohamed NA (2011)		-
	Saleh (2013)	Psychosocial stressors	t =13.38;p=0.001^*^
	Alasoom (2014)		1.9 [1.11- 3.3]
	Al-fayumi-Zeadna (2015)		1.7 [0.9–3.4]
Social and family support	Chaaya (2002)	Good support	0.66 [0.35-1.26]
	Mohammad KI (2011)	Low support	-
	Mohamed NA (2011)		-
	Bener (2012)	Low support	1.52 [1.0-2.14]
	Al-Modayfer (2015)	Family violence	2.55 [1.14- 5.71]
Major financial problems	Alami (2006)		0.49 [0.16-1.46]
	Bener (2012)		2.37 [1.56-3.58]
	Mohammed ES (2014)		-

* mean difference using t-test.

## LIST OF ABBREVIATIONS

PPD: = Postpartum Depression.
MDE: = Major Depressive Episode.
APA: = American Psychiatric Association
MCHC: = Maternal and Child Health Clinic.
PHCH: = Primary Health Care Clinic.
EPDS: = Edinburgh Postnatal Depression Scale
BDI: = Beck Depression Inventory.
DASS: = Depression, Anxiety and Stress Scale
MINI: = Mini International Neuropsychiatric Interview.
PSE: = Present State Examination
SES: = Socio-economic Status
CS: = Caesarean Section.
OR: = Odds Ration
CI: = Confidence Interval.
